# Supplementary material for: Isolation and Characterization of Two Pseudorabies Virus and Evaluation of Their Effects on Host Natural Immune Responses and Pathogenicity
Source: Viruses. 2022 Mar 29;14(4):712. doi: 10.3390/v14040712 (PMC9032386; doi:10.3390/v14040712)
Supplement: Supplementary file 1 [file viruses-14-00712-s001.zip › viruses-1620184-Supplementary Materials.pdf]

viruses

MDPI

Supplementary Materials

Isolation and Characterization of Two Pseudorabies Virus and Evaluation of Their Effects on Host Natural Immune Responses and Pathogenicity

Qiongqiong Zhou [1,†], Longfeng Zhang [1,2,†], Hongyang Liu [1], Guangqiang Ye [1], Li Huang [1,3,*] and Changjiang Weng [1,3,*]

Supplemental Materials and Methods

Cells and Viruses

PAMs and mouse peritoneal macrophages, isolated from the 4-week-old healthy specific pathogen free (SPF) piglets and 6-week-old healthy SPF mice as previously described [1,2], were maintained in RPMI-1640 medium containing 10% fetal bovine serum (FBS, Gibco). THP-1, PK-15 and HEK293T cells obtained from ATCC were cultured respectively in RPMI-1640 and Dulbecco's Modified Eagle's medium (DMEM) supplemented with 10% FBS. All the cells were maintained at 37 °C with 5% CO_2. PRV-TJ strain (GenBank number: KJ789182) is kindly provided by Prof. Tongqing An (Harbin Veterinary Research Institute of Chinese Academy of Agricultural Sciences, China), which is a high-virulent variant belonging to genotype 2.2. PRV-GD and PRV-JM were recently isolated from two different pig farms as previous described [3].

PCR Detecting gB and gE Genes during PRV Isolation

PRV *gB* and *gE* genes were detected by PCR amplification to confirm the existence of PRV in cell culture. Based on PRV-TJ strain, the primers for *gB* and *gE* detection were designed and showed in Table S2. PCR was performed and the products were analyzed by 1% agarose gel electrophoresis and visualized ultraviolet light.

$TCID_{50}$

The $TCID_{50}$ assay was performed as previously described [3]. Briefly, PK-15 cells grown in a 96-well plate were infected by PRV strains with 0.1 mL/well of 10-fold serially diluted supernatants in quintuplicate. After incubating for 2 h at 37 °C, unattached virus was removed, and DMEM supplemented with 2% FBS was added to the PK-15 cells. Five days post-infection, the $TCID_{50}$ was determined by the Reed-Muench method. All data are shown as the means of three independent experiments.

One-Step Growth Analysis

The one-step growth curves of PRV-TJ strain, PRV-GD and PRV-JM isolates were determined in PK-15 cells, PAMs, THP-1 and mouse peritoneal macrophages, as previously described [4]. Briefly, the confluent monolayer cells in 24-well plates were inoculated with PRV strains in triplicates at multiplicity of infection (MOI) of 0.1 at 37 °C for 2 h. Subsequently, both cells and the supernatant were harvested at 0, 12, 24, 36 and 48 h, respectively. After three freeze-thaw cycles, the cellular debris was removed by centrifugation at 5000 rpm for 20 min and the supernatant was titrated as $TCID_{50}$ in PK-15 cells. Results were presented as Mean ± SEM from three replicates.

RNA Extraction and Quantitative PCR (qPCR)

Total RNA was extracted from cells and tissue homogenates using TRIzol reagent (Invitrogen), and the reverse transcription products were amplified using the Agilent-Strata gene MxReal-Time qPCR system with a PrimeScript™ RT Reagent Kit (Takara). The

mRNA levels of IL-1β and other indicated genes were tested using an Agilent-Strata gene MxReal-Time qPCR system with TB Green® Premix ExTaq™ II (TliRNaseH Plus) (Takara) according to the manufacturer's instructions. The primers were listed in Table S3. The mRNA levels of genes were normalized to GAPDH. The final mRNA levels of genes in this study were calculated using the comparative cycle threshold method.

gB-Specific Absolute Quantification PCR

Genomic DNA was extracted from the tissues or EDTA-treated whole peripheral blood by using Universal Genomic DNA Kit (AXYGEN) and applied for *gB*-specific absolute quantification PCR to precisely detect viral copies. The primers and TaqMan probe sequences were listed in Table S4. The final virus copies were calculated based on the standard curve established with the recombinant pCAGGS-Flag-*gB* plasmid as the template, and presented as Mean ± SEM from three replicated wells.

ELISA Assay

The concentrations of IL-1β (mouse, ABclonal; pig, R&D systems), IL-6 (mouse, ABclonal; pig, R&D systems), TNF-α (mouse, ABclonal), IFN-α (pig, RayBio) and IFN-β (mouse, NOVUS Biologicals; pig, abcam) in cell culture supernatants and serum were measured by ELISA kits according to the manufacturer's instructions.

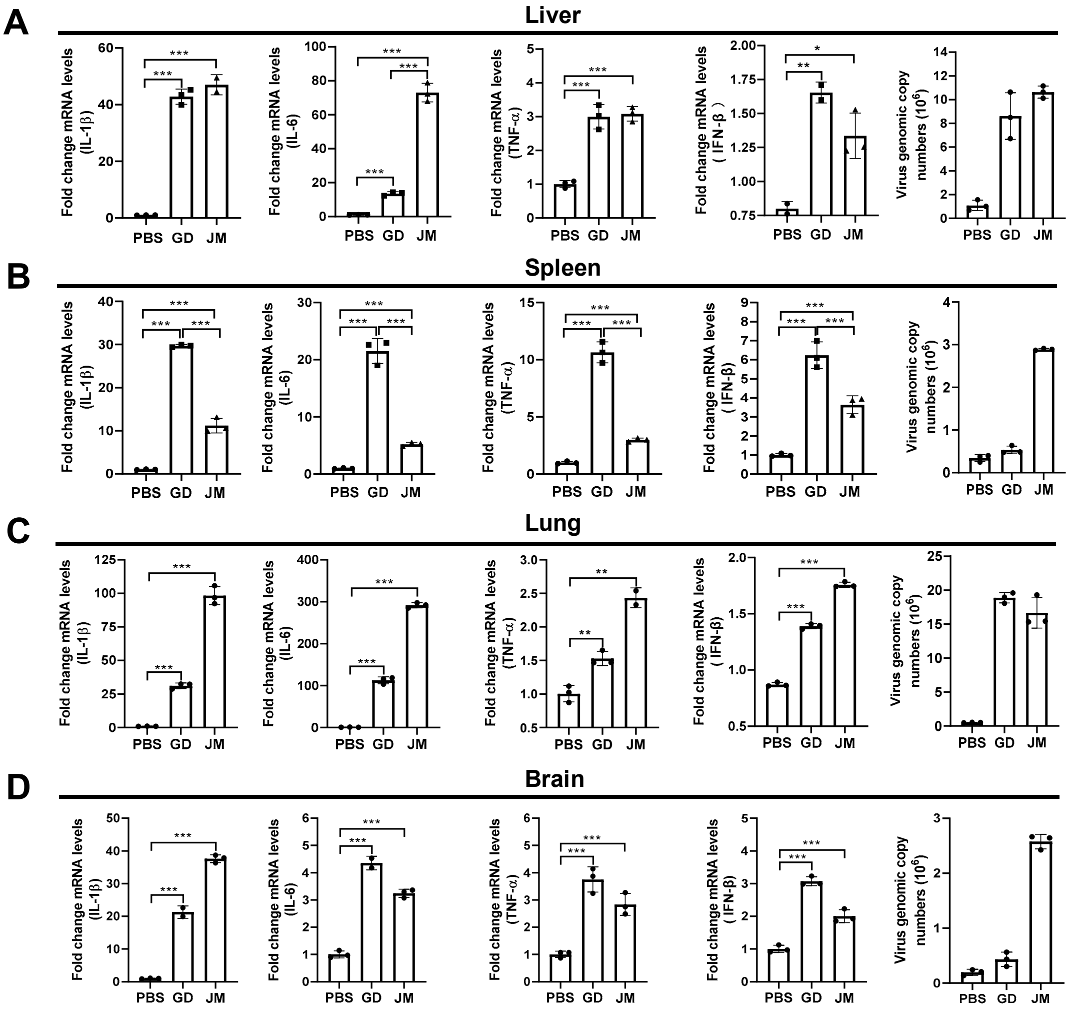

Figure S1. qPCR analysis of the mRNA level of IL-1β, IL-6, TNF-α, IFN-β and PRV genomic copy number in the liver (A), spleen (B), lung (C) and brain (D) from the mice challenged by PRV-GD

and PRV-JM. The significance of differences was analyzed with *t* test. * $p < 0.05$, ** $p < 0.01$, *** $p < 0.001$.

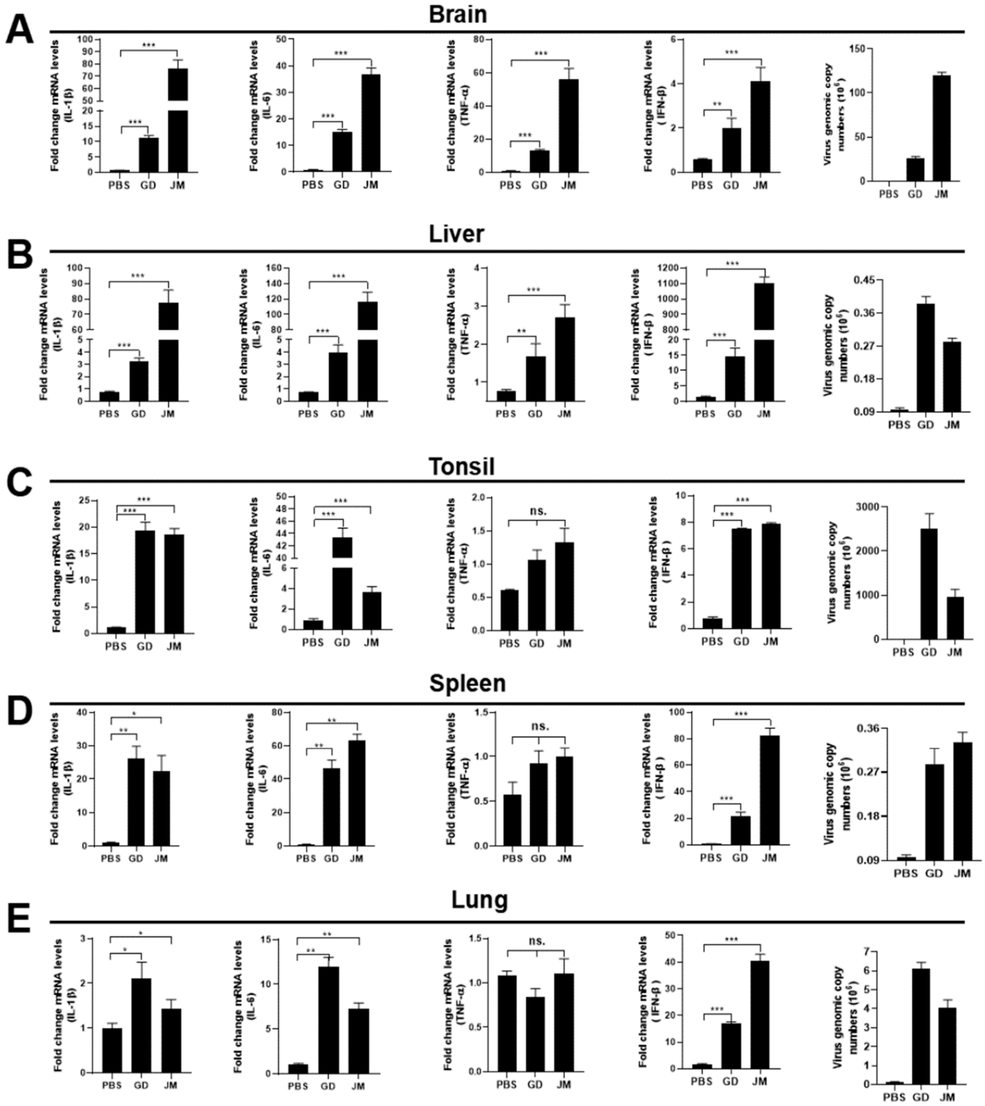

Figure S2. qPCR analysis of the mRNA level of IL-1β, IL-6, TNF-α, IFN-β and PRV genomic copy number in the brain (A), liver (B), tonsil (C), spleen (D) and lung (E) from the piglets challenged by PRV-GD or PRV-JM isolates. The significance of differences were analyzed with *t* test. * $p < 0.05$, ** $p < 0.01$, *** $p < 0.001$, ns., no significant difference.

Table S1. Detection the antibody level of PRV-gB protein in antisera.

	Antisera From PRV-Ea Vaccination						PRV-JM-Infection Antisera			
Number	M2	M5	M6	M9	M13	M18	06	92	93	94
S/N	0.0501	0.0508	0.0520	0.0545	0.0532	0.0619	0.1675	0.1718	0.2337	0.2168

Note: Samples were regarded as positive if they had an S/N ratio ≦ 0.6 and negative if it had an S/N ration ≥ 0.6.

Table S2. Primers used in this study for the PRV detection.

Primer Name	Sequence (5′ → 3′)	Product Size (bp)
PRV-*gB*-F	GTGCTCTTCAAGGAGAACATCGCCCCGCAC	790
PRV-*gB*-R	CCAGGTGCACGCGCTGCAGGTCGAGCTGCG	
PRV-*gE*-F	TTCGGCTCGGCCCTCGCATCCCTGAGGGAG	870
PRV-*gE*-R	GTTGTGGGTCATCACGAGCACGTACAGCCC	

Table S3. Primers used for RT-qPCR amplification.

Primer Name	Sequence (5′ → 3′)
m-GAPDH-F	TGGCCTTCCGTGTTCCTAC
m-GAPDH-R	GAGTTGCTGTTGAAGTCGCA
m-IL-1β-F	GAAATGCCACCTTTTGACAGTG
m-IL-1β-R	TGGATGCTCTCATCAGGACAG
m-IL-6-F	ACAAAGCCAGAGTCCTTCAGA
m-IL-6-R	TCCTTAGCCACTCCTTCTGT
m-TNF-α-F	ACTGAACTTCGGGGTGATCG
m-TNF-α-R	TCTTTGAGATCCATGCCGTTG
m-IFN-β-F	CTGCGTTCCTGCTGTGCTTC
m-IFN-β-F	CGCCCTGTAGGTGAGGTTGAT
p-GAPDH-F	ATCACTGCCACCCAGAAGACT
p-GAPDH-R	CATGCCAGTGAGCTTCCCGTT
p-IL-1β-F	GCTGGAGGATATAGACCCC
p-IL-1β-R	GTTGGGGTACAGGGCAGAC
p-IL-6-F	CTGCTTCTGGTGATGGCTACTG
p-IL-6-R	GGCATCACCTTTGGCATCTT
p-TNF-α-F	ACCACGCTCTTCTGCCTACTGC
p-TNF-α-R	TCCCTCGGCTTTGACATTGGCTAC
p-IFN-β-F	AGCACTGGCTGGAATGAAACCG
p-IFN-β-R	CTCCAGGTCATCCATCTGCCCA

Table S4. The primers and TaqMan probe sequences [1,2].

Primer Name	Sequence (5′ → 3′)
gB-probe-F	ACGGCACGGGCGTGATC
gB-probe-R	ACTCGCGGTCCTCGAGCA
gB-TaqMan-probe	FAM-CTCGCGCGACCTCATCGAGCCCTGCAC-MGB

Reference

1. Zhang, K.; Zhang, Y.; Xue, J.; Meng, Q.; Liu, H.; Bi, C.; Li, C.; Hu, L.; Yu, H.; Xiong, T.; et al. DDX19 Inhibits Type I Inter-feron Production by Disrupting TBK1-IKKε-IRF3 Interactions and Promoting TBK1 and IKKε Degradation -ScienceDirect. *Cell Rep.* **2019**, *26, 1258–1272.*
2. Zhou, Q.; Yan, B.; Sun, W.; Chen, Q.; Shi, D. Pig Liver Esterases Hydrolyze Endocannabinoids and Promote Inflammatory Response. *Front. Immunol.* **2021**, *12*, 670426.
3. Qingyun, L.; Xiaojuan, W.; Caihua, X.; Shifang, D.; Hongna, Y.; Shibang, G.; Jixuan, L.; Lingzhi, Q.; Fuguo, B.; Dongfang, W.A.; et al. A Novel Human Acute Encephalitis Caused by Pseudorabies Virus Variant Strain. *Clin Infect Dis*. **2021**, *73*, e3690–e3700.
4. Luo, Y.; Li, N.; Cong, X.; Wang, C.H.; Du, M.; Li, L.; Zhao, B.; Yuan, J.; Liu, D.D.; Li, S. Pathogenicity and genomic characterization of a pseudorabies virus variant isolated from Bartha-K61-vaccinated swine population in China. *Veter- Microbiol.* **2014**, *174*, 107–115. doi:10.1016/j.vetmic.2014.09.003.
